# The complete mitochondrial genome of *Sargassum siliquastrum* (Phaeophyceae) and its phylogenetic analysis

**DOI:** 10.1080/23802359.2020.1829134

**Published:** 2020-10-21

**Authors:** Jingjing Li, Huan Li, Yuanxin Bi

**Affiliations:** a Key Laboratory of Marine Hazards Forecasting, Ministry of Natural Resources, Hohai University, Nanjing, China; b Key Laboratory of Sustainable Utilization of Technology Research for Fishery Resource of Zhejiang Province, Marine Fisheries Research Institute of Zhejiang, Zhoushan, China

**Keywords:** *Sargassum siliquastrum*, Sargassaceae, phylogenetic analysis, mitochondrial genome

## Abstract

The complete *S. siliquastrum* mitogenome length was 34,765 bp. The mitogenome contains 67 genes, including 37 protein-coding, three rRNA, 25 tRNA genes, and two conserved open reading frames (ORFs). The overall GC content of the genome is 36.59%. The complete mitogenome sequence provided herein would help understand *Sargassum* evolution.

*Sargassumsiliquastrum* (Mertens ex Turner) C. Agardh belongs to the Sargassaceae family of Fucales, Phaeophyta. It occurs widely in temperate waters in Japan (Terada et al. 2019), Korea (Lee et al. 2013) and China (Huang et al. 2017). *Sargassum siliquastrum* forests have experienced a noticeable regression because of habitat destruction and ocean warming (Leung et al. 2014). In this study, we obtained the complete mitochondrial genome of *S. siliquastrum*. This new information would provide better understanding of mitochondrial genome diversity in the Fucales and contribute to the preservation of its genetic resources.

The specimen was collected from Nanhuangcheng Island (38°22′12″N; 120°54′24″E) on 13 January 2020, and stored at the Marine Biological Museum, Chinese Academy of Sciences with an accession number MBM286789. Mitochondrial DNA was extracted using a Plant Tissue Mitochondrial DNA Extraction Kit (Genmed Scientific Inc., Arlington, MA, USA) according to the manufacturer’s instructions. Illumina pair-end library would be used for Illumina NovaSeq 6000 sequencing (Shanghai BIOZERON Co., Ltd). In addition, whole genome shotgun libraries were generated and sequenced on a Pacific Biosciences RS instrument. High quality data, ca. 10.43 Gb were obtained. De novo assembly and confirmation were conducted by ABySS (http://www.bcgsc.ca/platform/bioinfo/software/abyss, v2.0.2). The transfer RNA (tRNA) genes were predicted by tRNAscan-SE and the ribosomal RNA (rRNA) genes and protein coding genes were used to make homology alignments with *Sargassum yezoense* (NC038156) as reference genome. In order to better elucidate the phylogenetic relationship of *S. siliquastrum* and its relative species, 15 mitochondrial genome sequences were downloaded from the GenBank database. Maximum-likelihood (ML) tree was constructed using the selected model (GTR + G + I) with 100 bootstrap replicates in PhyML 3.0 (Guindon et al. 2010). *Turbinaria ornata* (GenBank accession number KM501562) was chosen as the out-group.

The complete (MT712045) mitogenome comprises a circular DNA molecule measuring 34,765 bp in length with 36.59% overall GC content. The mitogenome contains 67 genes, including 37 protein-coding, 3 rRNA, 25 tRNA genes, and 2 conserved open reading frames (ORFs). All protein-coding genes use the start codon ATG. 27 protein-encoding genes used TAA as a stop codon and the remaining were TAG (5) and TGA (5). The lengths of three rRNA genes are 1085 bp (rnl rRNA), 873 bp (rns rRNA), and 122 bp (rrn5). All genes show the typical gene arrangement conforming to the Sargassaceae family consensus. ML tree showed that *S. siliquastrum* clustered together with *Sargassum yezoense* (Figure 1). The complete mitogenome sequence provided herein would help understand phylogenetic evolution of *S. siliquastrum.*

**Figure 1.**
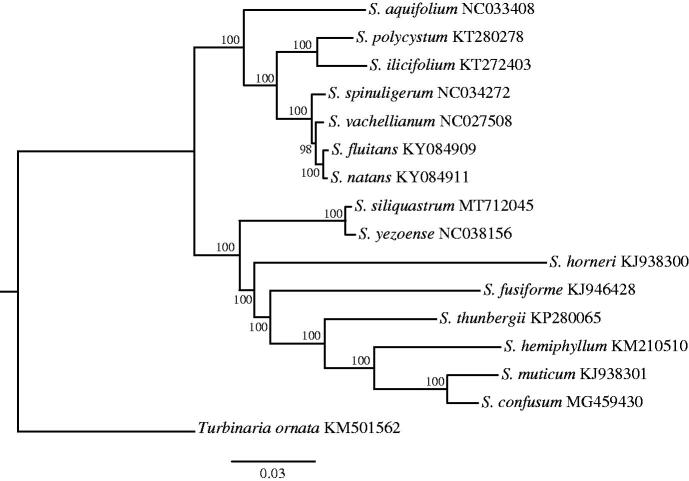
Maximum-likelihood phylogenetic tree based on *S. siliquastrum* and 15 other mitochondrial genomes of Sargassaceae family in the NCBI.

## Data Availability

The data that support the findings of this study are openly available in repository under Accession: MT712045. https://www.ncbi.nlm.nih.gov/nuccore/MT712045
